# A homogenized approach to classify advanced gastric cancer patients with limited and adequate number of pathologically examined lymph nodes

**DOI:** 10.1186/s40880-019-0370-z

**Published:** 2019-06-10

**Authors:** Sharvesh Raj Seeruttun, Lipu Xu, Fangwei Wang, Xiaodong Yi, Cheng Fang, Zhimin Liu, Wei Wang, Zhiwei Zhou

**Affiliations:** 1 Department of Gastric Surgery, State Key Laboratory of Oncology in South China, Collaborative Innovation Center for Cancer Medicine, Sun Yat-sen University Cancer Center, 651 Dongfeng Road East, Guangzhou, 510060 Guangdong P. R. China; 2 Department of Surgical Oncology, Affiliated Lu’an Hospital of Anhui Medical University, Lu’an, 237005 Anhui P. R. China

**Keywords:** Advanced gastric cancer, Limited lymph nodes, Adequate lymph nodes, R0 gastrectomy, AJCC, Eighth edition, Modified classification, Akaike information criterion, Lymph node ratio, Prognosis

## Abstract

**Background:**

The prognosis of gastric cancer patients with a limited number of pathologically examined lymph nodes (eLN, < 16) is dismal compared to those with adequately eLN (≥ 16), yet they are still classified within the same subgroups using the American Joint Committee on Cancer (AJCC) staging system. We aimed at formulating an easy-to-adopt and clinically reliable stratification approach to homogenize the classification for these two categories of patients.

**Methods:**

Patients staged according to the 8th AJCC pathological nodal (N) and tumor-node-metastasis (TNM) classification were stratified into a Limited and Adequate eLN cohort based on their number of pathologically examined LNs. The statistical differences between the 5-year overall survival (OS) rates of both cohorts were determined and based on which, patients from the Limited eLN cohort were re-classified to a proposed modified nodal (N′) and TNM (TN′M) classification, by matching their survival rates with those of the Adequate eLN cohort. The prognostic performance of the N′ and TN′M classification was then compared to a formulated lymph-node-ratio-based nodal classification, in addition to the 8th AJCC N and TNM classification.

**Results:**

Significant heterogeneous differences in 5-year OS between patients from the Limited and Adequate eLN cohort of the same nodal subgroups were identified (all *P* < 0.001). However, no significant differences in 5-year OS were observed between the subgroups N0, N1, N2, and N3a of the Limited eLN cohort when compared with N1, N2, N3a, and N3b from the Adequate eLN cohort, respectively (*P* = 0.853, 0.476, 0.114, and 0.230, respectively). A novel approach was formulated in which only patients from the Limited eLN cohort were re-classified to one higher nodal subgroup, denoted as the N′ classification. This re-classification demonstrated superior stratifying and prognostic ability as compared to the 8th AJCC N and lymph-node-ratio classification (Akaike information criterion values [AIC]: 12,276 vs. 12,358 vs. 12,283, respectively). The TN′M classification also demonstrated superior prognostic ability as compared to the 8th AJCC TNM classification (AIC value: 12,252 vs. 12,312).

**Conclusion:**

The proposed lymph node classification approach provides a clinically practical and reliable technique to homogeneously classify cohorts of gastric cancer patients with limited and adequate number of pathologically examined lymph nodes.

## Introduction

Gastric cancer (GC) is the second most common cancer in China [1] and the third leading cause of cancer-related death worldwide [2]. Its high mortality rate can be largely attributed to the early spread of cancer cells via the lymphatic system [3]. As a result, the number of surgically retrieved lymph nodes (LNs) is positively correlated with the likelihood of complete removal of all perigastric metastatic LNs and is among the most determining factors for assessing the quality of gastrectomies [4, 5].

Studies have shown that patients with ≥ 16 pathologically examined LNs (eLNs) have better prognoses as compared to those with < 16 eLNs [6, 7]. The two major acknowledged bodies in the gastric oncological field, the National Comprehensive Cancer Network (NCCN) [8] and the American Joint Committee on Cancer (AJCC) [9], both advocate the retrieval of at least 16 LNs for optimizing the radicality of D2 lymphadenectomies and enabling proper staging of the disease.

However, performing such gastrectomies are challenging as they necessitate a high level of expertise due to the risky anatomical surrounding structures. In Asian countries (such as China, Japan and Korea), these are comparatively easier as high-volume institutions refer to centers performing at least 200 gastrectomies annually in which high-quality lymphadenectomies are routinely achieved [10], whereas in Western settings, these might prove arduous due to their smaller number of cases and where “high-volume” institutions often refer to centers with 15–20 GC cases per year [11, 12]. Correspondingly, an analysis of 18,043 GC patients from the Surveillance, Epidemiology, and End Results Program (SEER) database [13], studies from 691 hospitals in the United States [14] and 18 hospitals in the United Kingdom [15] found that only 33%, 40% and 31% of their respective gastrectomies met the minimum recommended number of surgically retrieved LNs.

In addition, patient-related factors such as elderly patients presenting with comorbidities [16], obese patients in whom adipose tissues often obscure surgical visibility for retrieval of LNs intermingled with major blood vessels [17–19] and/or surgery-related factors such as risky tumor locations (esophagogastric or gastro-duodenal junctions) and large tumor size may also complicate surgeries [20]. These commonly observed factors in the day-to-day practice may at times compel even experienced surgeons to opt for a limited number of LNs retrieved (< 16 eLNs).

Consequently, the worldwide annual total number of gastrectomies with limited eLNs may agglomerate to a substantial proportion, yet neither the AJCC nor the NCCN committees have proposed a standard classification for this category of patients. Clinically, this often leads to therapeutic confusion, coercing an unstandardized approach by oncologists with no other choice than to rely on their own clinical judgment for the counseling [21] and planning of treatments for this category of patients [22].

Thus, the aim of this study was to develop an easy-to-adopt and clinically reliable stratifying approach for homogenizing the nodal classification of patients with limited eLN in cohorts of GC patients comprising of both patients with limited and adequate number of pathologically eLNs.

## Methods

### Patient population

Upon approval from the ethics committee of the Sun Yat-sen University Cancer Center (Guangzhou, China), our prospectively collected database was examined from January 2000 to December 2012 for eligible patients with the following criteria: (1) no prior neoadjuvant therapy, (2) R0 gastrectomy irrespective of the type of lymphadenectomy, (3) absence of previous and/or synchronous malignancy, (4) postoperative histological confirmation of advanced gastric adenocarcinoma. Patients with tumors pathologically confirmed as not invading deeper than the submucosa (pT1) of the gastric wall were excluded. All patients’ follow-up adhered to the NCCN GC guidelines [8]; in brief, regular outpatient visits for complete physical examination, routine blood chemistry and radiological examinations (chest X-ray or computed tomography of the chest and abdomen) every 3 to 6 months for the first 2 years post-gastrectomy, every 6 to 12 months for the following 3 to 5 years and on an annual basis thereafter. The last date of follow-up was June 30, 2018. All participants in this study provided consent forms for participation.

### Stratification of patients

Initially, all patients, irrespective of their number of eLNs, were classified according to the 8th AJCC GC pathological nodal classification (pN) and labeled as the Combined eLN cohort. Then, those with < 16 and ≥ 16 pathologically eLNs were stratified as the Limited and Adequate eLN cohort, respectively.

### Formulating a novel approach for nodal classification

First, we analyzed the 5-year overall survival (OS) rate of patients in the Limited, Adequate and Combined eLN cohort. Second, the statistical difference in 5-year OS of the corresponding nodal subgroups of the Limited and Adequate eLN cohort was determined (i.e., N0–N3b of the Limited eLN cohort vs. N0–N3b of the Adequate eLN cohort, respectively). Third, based on the obtained difference in statistics, a modified nodal classification (N′) was formulated using a similar analogy as with the 8th AJCC GC pN classification (i.e. using same nodal subgroups classification but under different group names).

Next, to further assess the stratifying efficacy of the N′ classification, we also determined the lymph-node-ratio (LNR) of the Combined eLN cohort for referential comparison, as LNR is one of the most widely investigated alternate nodal classifications in GC, using methods described in our previous study [23]. Further, to optimize its comparison with our proposed N′ and the 8th AJCC pN classification, the best cut-point ranges of LNR for 5 nodal subgroups were computed, found as being 0.000–0.020, 0.021–0.100, 0.110–0.300, 0.310–0.500 and 0.510–1.000 and were denoted as LNR 1, LNR 2, LNR 3, LNR 4 and LNR 5, respectively. To concisely investigate the clinical applicability of the N′ classification, we determined the prognostic performance of the proposed N′ classification in comparison to the 8th AJCC pN classification and the LNR by investigating their likelihood χ^2^, linear trend χ^2^ and Akaike information criterion values (AIC).

Further, we substituted the N subgroups of the 8th AJCC pathological GC edition with our N′ classification to develop the tumor-modified node-metastasis (TN′M) classification. The stratifying and prognostic performance of the TN′M system was then compared with those of the 8th AJCC GC pTNM edition, using the above-mentioned statistics. In this study, all the stages concerning the different classifications of LN (N, N′ and LNR), depth of tumor invasion (T) and tumor-node-metastasis (TNM and TN′M) were derived from the 8th AJCC GC pathological classification.

### Statistical analysis

Analysis for the best LNR cut-points was investigated using X-tile software (https://medicine.yale.edu/lab/rimm/research/software.aspx, version 3.6.1, Rimm Lab, Yale School of Medicine, BML112, New Haven, CT). OS time was defined as the time from the date of surgery until the last follow-up time or date of tumor-related death. The Kaplan–Meier method was used for all survival analyses. The Cox proportional hazard model with forward stepwise regression was used to compute three separate multivariate analyses, namely, multivariate 1, 2 and 3, which consisted of parameters found to be significant (*P* < 0.05) in univariate analysis of the 8th AJCC pN classification, LNR, and N′ classification, respectively. To compare the homogeneity (difference in survival time among patients within the same subgroup of a staging system), discriminatory ability (difference in survival time among patients of different subgroups of a staging system), and overall prognostic performance of the different staging systems, the likelihood ratio χ^2^ test, linear trend χ^2^ test, and AIC were computed, respectively. The AIC statistics were defined using the following equation: AIC = − 2log maximum likelihood + (2 × the number of parameters in the model), in which stronger overall prognostic performance of the investigated classification corresponded to a smaller AIC value [23].

Statistical analyses were performed using SPSS software (version 21.0, SPSS Inc., Chicago, IL) and R statistical software (version 3.3.1, the R Foundation for Statistical Computing, Vienna, Austria). A *P*-value < 0.05 (2-sided) was considered as statistically significant.

## Results

### Patient characteristics and their association with OS

A total of 2304 patients with advanced GC were included. The patients’ age of the Combined eLN cohort ranged from 18 to 89 years (median, 59 years), and of the fourteen factors analyzed (Table 1), only gender showed no correlation with OS in univariate analysis (*P* = 0.956). The clinical parameters found to be independently associated with OS on multivariate analyses (favorable characteristics in parentheses; Table 2) were as follows: age (≤ 60 years), tumor location (lower third of stomach), Lauren type (intestinal), partial gastrectomy, depth of tumor infiltration (pT2), and classification of metastatic LNs (pN, LNR and N′). However, when considering the categorization of eLNs, no independent correlation of the N′ classification to OS was found (*P* = 0.374).

**Table 1:** Association of patient clinicopathological characteristics with overall survival

Characteristic	Total [cases (%)]	5-year OS (%)	HR (95% CI)	*P*-value
Gender				0.956
Male	1603 (69.6)	57.2	Ref.	
Female	701 (30.4)	56.8	1.004 (0.700–1.158)	
Age (years)				< 0.001
≤ 60	1226 (53.2)	61.1	Ref.	
> 60	1078 (46.8)	52.5	1.316 (1.154–1.501)	
Tumor location				< 0.001
Lower 1/3	839 (36.4)	67.9	Ref.	
Middle 1/3	393 (17.1)	56.9	1.450 (1.184–1.776)	
Upper 1/3	967 (42.0)	51.0	1.720 (1.471–2.010)	
> 1/3 of stomach	105 (4.6)	25.4	3.581 (2.723–4.708)	
Tumor size (cm)				< 0.001
≤ 4.5	1180 (51.2)	64.8	Ref.	
> 4.5	1124 (48.8)	49.0	1.600 (1.401–1.827)	
Lauren type				< 0.001
Intestinal	804 (34.9)	62.9	Ref.	
Diffuse	1500 (65.1)	54.0	1.364 (1.182–1.575)	
Type of gastrectomy				< 0.001
Partial	1844 (80.0)	60.3	Ref.	
Total	460 (20.0)	41.4	1.721 (1.471–2.014)	
Examined lymph nodes				0.003
< 16	794 (34.5)	53.3	Ref.	
≥ 16	1510 (65.5)	59.3	0.817 (0.715–0.934)	
8th AJCC pT classification				< 0.001
T2	297 (12.9)	85.5	Ref.	
T3	492 (21.4)	65.5	2.203 (1.578–3.074)	
T4a	1296 (56.3)	51.5	3.588 (2.666–4.831)	
T4b	219 (9.5)	35.3	5.717 (4.087–7.996)	
8th AJCC pN classification				< 0.001
N0	653 (28.3)	82.7	Ref.	
N1	434 (18.8)	65.2	2.192 (1.705–2.818)	
N2	491 (21.3)	49.4	3.651 (2.906–4.589)	
N3a	489 (21.2)	37.8	5.078 (4.050–6.367)	
N3b	237 (10.3)	24.8	7.684 (6.001–9.839)	
LNR classification				
LNR 1	666 (28.9)	82.5	Ref.	< 0.001
LNR 2	285 (12.4)	72.5	1.682 (1.246–2.270)	
LNR 3	499 (21.7)	57.3	2.830 (2.237–3.580)	
LNR 4	360 (15.6)	42.1	4.371 (3.458–5.526)	
LNR 5	494 (21.4)	25.4	7.465 (6.004–9.281)	
N′ classification				< 0.001
N′0	382 (16.6)	87.5	Ref.	
N′1	498 (21.6)	75.1	2.314 (1.612–3.321)	
N′2	489 (21.2)	59.1	4.348 (3.080–6.137)	
N′3a	591 (25.7)	40.4	7.170 (5.138–10.006)	
N′3b	344 (14.9)	23.1	12.713 (9.046–17.866)	
8th AJCC pTNM classification				< 0.001
IB	162 (7.0)	94.8	Ref.	
IIA	202 (8.8)	85.3	2.353 (1.175–4.710)	
IIB	476 (20.7)	75.5	3.830 (2.060–7.123)	
IIIA	674 (29.3)	54.7	8.168 (4.473–14.915)	
IIIB	501 (21.7)	38.8	12.800 (6.999–23.412)	
IIIC	289 (12.5)	23.2	20.845 (11.338–38.322)	
TN′M classification				< 0.001
IB′	105 (4.6)	96.9	Ref.	
IIA′	178 (7.7)	90.7	2.845 (0.972–8.324)	
IIB′	362 (15.7)	77.8	5.103 (1.860–14.000)	
IIIA′	685 (29.7)	64.6	9.839 (3.662–26.434)	
IIIB′	569 (24.7)	42.2	18.918 (7.052–50.750)	
IIIC′	405 (17.6)	22.4	34.795 (12.961–93.409)	

*OS* overall survival, *HR* hazard ratio, *CI* confidence interval, *AJCC* American Joint Committee on Cancer, *pN* pathologically examined nodal classification, *N*′ pathologically examined modified nodal classification, *pTNM* pathological tumor-node-metastasis classification, *TN*′*M* pathological tumor-modified node-metastasis classification, *LNR* lymph-node-ratio, *Ref.* reference

**Table 2:** Multivariate analyses of factors associated with 5-year overall survival for the 8th AJCC pN, LNR and N′ classifications

Characteristics	Multivariate analysis 1^a^				Multivariate analysis 2^b^				Multivariate analysis 3^c^		
	HR	95% CI	*P* value		HR	95% CI	*P* value		HR	95% CI	*P* value
Age (years)											
≤ 60	Ref.				Ref.				Ref.		
> 60	1.404	1.226–1.607	< 0.001		1.392	1.216–1.592	< 0.001		1.401	1.225–1.604	< 0.001
Tumor location											
Lower 1/3	Ref.				Ref.				Ref.		
Middle 1/3	1.264	1.006–1.590	0.045		1.259	1.000–1.586	0.050		1.268	1.009–1.593	0.042
Upper 1/3	1.666	1.406–1.973	< 0.001		1.551	1.309–1.837	< 0.001		1.691	1.432–1.997	< 0.001
Stomach > 1/3	1.737	1.233–2.446	0.002		1.735	1.231–2.446	0.002		1.738	1.235–2.445	0.002
Lauren type											
Intestinal	Ref.				Ref.				Ref.		
Diffuse	1.229	1.057–1.430	0.007		1.236	1.063–1.438	0.006		1.222	1.051–1.421	0.009
Gastrectomy											
Partial	Ref.				Ref.				Ref.		
Total	1.262	1.024–1.556	0.029		1.339	1.085–1.652	0.007		1.231	1.000–1.525	0.050
Examined LNs											
< 16	Ref.				Ref.				Ref.		
≥ 16	0.543	0.464–0.636	< 0.001		0.861	0.746–0.994	0.041		NA^#^	NA^#^	0.374
8th AJCC pT classification											
T2	Ref.				Ref.				Ref.		
T3	1.428	1.020–2.000	0.038		1.467	1.047–2.055	0.026		1.439	1.028–2.015	0.034
T4a	2.063	1.524–2.793	< 0.001		2.094	1.546–2.835	< 0.001		2.064	1.524–2.794	< 0.001
T4b	2.648	1.873–3.745	< 0.001		2.663	1.883–3.765	< 0.001		2.654	1.878–3.751	< 0.001
8th AJCC pN classification				LNR classification				N′ classification			
N0	Ref.			LNR 1	Ref.			N′0	Ref.		
N1	2.044	1.587–2.633	< 0.001	LNR 2	1.703	1.256–2.309	0.001	N′1	2.142	1.491–3.076	< 0.001
N2	3.328	2.638–4.198	< 0.001	LNR 3	2.717	2.142–3.446	< 0.001	N′2	3.746	2.650–5.295	< 0.001
N3a	5.874	4.629–7.455	< 0.001	LNR 4	3.861	3.042–4.900	< 0.001	N′3a	6.078	4.344–8.503	< 0.001
N3b	9.985	6.810–11.854	< 0.001	LNR 5	6.344	5.068–7.940	< 0.001	N′3b	10.419	7.373–14.724	< 0.001

*HR* hazard ratio, *CI* confidence interval, *LNs* lymph nodes, *AJCC* American Joint Committee on Cancer, *pT* pathological tumor depth classification, *pN* pathological nodal classification, *LNR* lymph node ratio, *N*′ pathological modified nodal classification, *Ref.* reference; *NA* not available

^a^Multivariate analysis 1: Clinicopathological factors showing significance in univariate analysis and the 8th AJCC pN classification, excluding the LNR classification and the N′ classification

^b^Multivariate analysis 2: Clinicopathological factors showing significance in univariate analysis and the LNR classification, excluding the 8th AJCC pN classification and the N′ classification

^c^Multivariate analysis 3: Clinicopathological factors showing significance in univariate analysis and the stages of the N′ classification, excluding the 8th AJCC pN classification and the LNR classification

^#^NA: *P* > 0.05, the corresponding HR and 95% CI values were not available

A total of 50,501 LNs from the 2304 investigated advanced GC patients were pathologically examined, of which 13,506 (26.7%) contained metastases. Overall, a median of 20 LNs (range, 1–79 LNs) were examined per patient. In the Limited eLN cohort (patients, *n* = 794, 34.5%), of the 7666 eLNs (median, 10; range, 1–15 LNs) there were 2166 (28.3%) metastatic LNs (median, 2; range, 1–15 LNs), while in the Adequate eLN cohort (patients, *n* = 1510, 65.5%), of the total 42,835 eLNs (median, 26; range, 16–79 LNs) there were 11,340 (26.5%) metastatic LNs (median, 5; range, 1–70 LNs).

Regarding the adverse clinical factors negatively suppressing the patients’ prognoses, of the 794 patients in the Limited eLN cohort (in parentheses, vs. annotate comparative proportion of patients in the Adequate eLN cohort), 55.0% of the investigated patients were older than 60 years (vs. 42.5%; *P* < 0.001), 61.8% of the tumors were located in the upper third of the stomach (vs. 31.5%; *P* < 0.001), 47.5% of the patients had tumors greater than 4.5 cm (vs. 49.5%; *P* = 0.381), and 67.7% had tumors that infiltrated the sub-serosal and serosal layers (vs. 64.7%; *P* = 0.144). These data demonstrate that patients in the Limited eLN cohort were comparatively older and had a larger proportion of proximal gastric tumors.

### Formulating a homogenized nodal classification for the Limited and Adequate eLN cohort

**Fig. 1:**
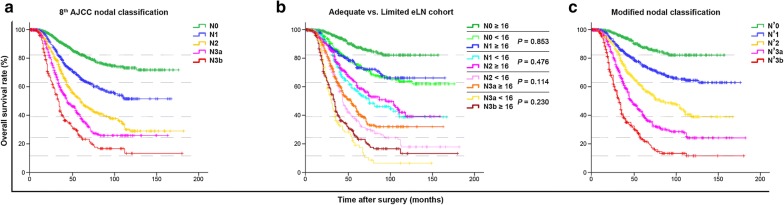
Kaplan–Meier analysis showing the detailed survival differences of the study cohort using the different nodal classifications. Illustration of the overall survival of advanced gastric cancer patients using the **a** 8th AJCC N classification, **b** 8th AJCC N classification stratified into Limited (< 16 eLNs) and Adequate (≥ 16 eLNs) eLN cohort, and **c** the N′ classification, which re-classified the patients from the Limited eLN cohort only more homogenously based on their statistical differences in overall survival with patients from the Adequate eLN cohort. Note: the horizontal broken lines demonstrate the survival differences between **c** and **a** and simultaneously the rationale for formulating **c** from **b**. *AJCC* American Joint Committee on Cancer; *N* nodal, *eLN* pathologically examined lymph node, *N*′ modified nodal

Figure 1a illustrates a clear demarcation between the OS curves of each nodal subgroup of the Combined eLN cohort using the 8th AJCC pN classification. Further stratified analysis revealed significant heterogeneous differences in the 5-year OS between the corresponding nodal subgroups of the Limited and Adequate eLN cohort (all *P* < 0.001; Table 3). Most importantly, the 5-year OS of patients in the nodal subgroups N0–N3a of the Limited eLN cohort were not only significantly inferior with their corresponding nodal subgroups N0–N3a from the Adequate eLN cohort but additionally approximated those of subgroups N1–N3b in the Adequate eLN cohort, respectively, without any significant statistical differences (all *P* > 0.05; Fig. 1b). We thereby formulated a novel and practical approach in which the N0, N1, N2, and N3a patients from the Limited eLN cohort only were each upgraded to one higher nodal stage and denoted as N′1, N′2, N′3a, and N′3b, respectively. The classification of patients from the Adequate eLN cohort remained unchanged. As such, the N′0 subgroup comprised of only the N0 patients from the Adequate eLN cohort. From Fig. 1c, we can visualize that the N′ classification corrected the underestimation of survival of the N0 subgroup of the adequate eLN cohort and showed an improved separation between the survival curves for patients with greater number of LN metastases (N′2 to N′3b).

**Table 3:** Analysis of the 5-year overall survival rates of patients using the 8th AJCC pN classification stratified into Limited (< 16 eLNs) and Adequate (≥ 16 eLNs) eLN cohorts

Nodal classification	Limited eLN cohort		Adequate eLN cohort		P value (Limited vs. Adequate eLN cohort)	Combined eLN cohort	
	Number [cases (%)]	5-year OS (%)	Number [cases (%)]	5-year OS (%)		Number [cases (%)]	5-year OS (%)
8th AJCC pN							
N0	271 (34.1)	76.4	382 (25.3)	87.5	< 0.001	653 (28.3)	82.7
N1	207 (26.1)	57.1	227 (15.0)	73.4	< 0.001	434 (18.8)	65.2
N2	209 (26.3)	36.3	282 (18.7)	60.8	< 0.001	491 (21.3)	49.4
N3a	107 (13.5)	19.2	382 (25.3)	43.0	< 0.001	489 (21.2)	37.8
N3b			237 (15.7)	24.8		237 (10.3)	24.8
pN′							
N′0			382 (25.3)	87.5		382 (16.6)	87.5
N′1	271 (34.1)	76.4	227 (15.0)	73.4	0.853	498 (21.6)	75.1
N′2	207 (26.1)	57.1	282 (18.7)	60.8	0.476	489 (21.2)	59.1
N′3a	209 (26.3)	36.3	382 (25.3)	43.0	0.114	591 (25.7)	40.4
N′3b	107 (13.5)	19.2	237 (15.7)	24.8	0.230	344 (14.9)	23.1
8th AJCC pTNM							
IB	57 (7.2)	91.6	105 (7.0)	96.9	0.136	162 (7.0)	94.8
IIA	81 (10.2)	78.5	121 (8.0)	90.4	0.169	202 (8.8)	85.3
IIB	195 (24.6)	72.4	281 (18.6)	77.7	0.038	476 (20.7)	75.5
IIIA	305 (38.4)	42.9	369 (24.4)	66.1	< 0.001	674 (29.3)	54.7
IIIB	137 (17.3)	25.4	364 (24.1)	43.9	< 0.001	501 (21.7)	38.8
IIIC	19 (2.4)	8.4	270 (17.9)	24.3	0.016	289 (12.5)	23.2
pTN′M							
IB′			105 (7.0)	96.9		105 (4.6)	96.9
IIA′	57 (7.2)	91.6	121 (8.0)	90.4	0.677	178 (7.7)	90.7
IIB′	81 (10.2)	78.5	281 (18.6)	77.7	0.995	362 (15.7)	77.8
IIIA′	316 (39.8)	63.1	369 (24.4)	66.1	0.325	685 (29.7)	64.6
IIIB′	205 (25.8)	39.3	364 (24.1)	43.9	0.197	569 (24.7)	42.2
IIIC′	135 (17.0)	18.8	270 (17.9)	24.3	0.218	405 (17.6)	22.4

*eLNs* pathologically examined lymph nodes, *OS* overall survival rate, *AJCC* American Joint Committee on Cancer, *pN* pathologically examined nodal classification, *N*′ pathologically examined modified nodal classification, *pTNM* pathological tumor-node-metastasis classification, *TN*′*M* pathological tumor-modified node-metastasis classification

### Differences between the N′ and LNR classification

**Fig. 2:**
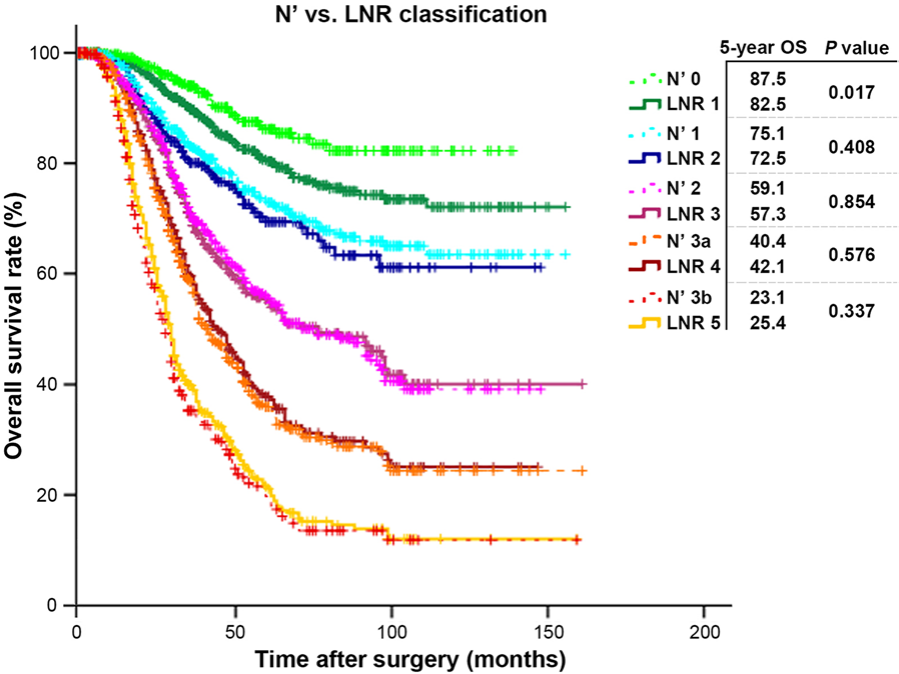
Juxtaposing the survival curves of the N′ classification with those of the LNR classification to illustrate the survival differences between these two nodal staging methods. *N*′ modified nodal, *LNR* lymph-node-ratio, *OS* overall survival rates

In contrast to the N′ classification, the LNR was observed to significantly underestimate the 5-year OS of patients with non-metastatic LNs (*P* = 0.017) and trends towards overestimating those subgroups of patients with higher rates of LN metastases (5-year OS of N′3a vs. LNR 4: 40.4% vs. 42.1% and 5-year OS of N′3b vs. LNR 5: 23.1% vs. 25.4%), although statistical significance was not reached (*P* = 0.576 and 0.337, respectively; Fig. 2). These data partly demonstrate that the N′ classification could accommodate a more diversified survival range (HR: 2.314–12.713 for N′1–N′3b vs. 1.682–7.465 for LNR2–LNR5) and provide a greater demarcation between patients with metastatic and non-metastatic LNs (Table 1).

### Prognostic performance of the N′ classification

Data from Table 1 illustrate that patients staged using the N′ classification had the widest range of 5-year OS (87.5%–23.1%) and HR values (2.314–12.713) in contrast to the LNR (5-year OS, 82.5%–23.1% and HR, 1.682–7.465) and the 8th AJCC pN classification (5-year OS, 82.7%–24.8% and HR, 2.192–7.684), which makes it more efficient at nodal subgroup stratification. In addition, the performance indices of the three nodal classifications listed in Table 4 show that the N′ classification has the highest likelihood ratio, signifying narrow differences in survival rates between patients within the same tumor stage and thus demonstrating the best homogeneity. Further, by demonstrating the lowest AIC value, the N′ classification proved to possess superior overall prognostic performance compared with the other competing nodal classifications.

**Table 4:** Performance indices of the different classifications

Classification	Likelihood ratio χ^2^ (homogeneity)	Linear trend χ^2^ (discriminatory ability)	AIC (overall performance)
8th AJCC pN	376.9	328.7	12,358
LNR	452.4	408.5	12,283
N′	458.7	390.0	12,276
8th AJCC pTNM	425.3	344.4	12,312
TN′M	485.1	400.6	12,252

*AJCC* American Joint Committee on Cancer, *pN* pathologically examined nodal classification, *LNR* lymph node ratio, *N*′ pathologically examined modified nodal classification, *pTNM* pathological tumor-node-metastasis classification, *TN*′*M* pathological tumor-modified node-metastasis classification, *AIC* Akaike information criterion

### Prognostic performance of the TN′M classification

**Fig. 3:**
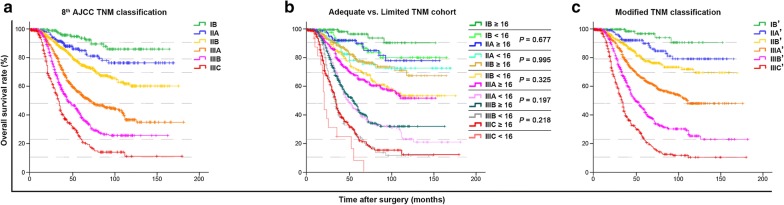
Kaplan–Meier analysis showing the detailed survival differences of the study cohort using the different TNM classifications. Illustration of the overall survival of advanced gastric cancer patients using the **a** 8th AJCC TNM classification, **b** 8th AJCC TNM classification stratified into Limited (< 16 eLNs) and Adequate (≥ 16 eLNs) eLN cohort, and **c** the TN′M classification, which consists of patients re-classified using the N′ classification and it can be found to demonstrate a better demarcation between patients with less advanced disease (IB′) in contrast to those with more advanced disease. Note: the horizontal broken lines demonstrate the survival differences between **c** and **a** and simultaneously the rationale for formulating **c** from **b**. *AJCC* American Joint Committee on Cancer, *TNM* tumor-node-metastasis, *eLN* pathologically examined lymph node, *TN*′*M* modified TNM

Figure 3a illustrate that although the survival curves for each stage of the Combined eLN cohort were well separated when using the 8th AJCC pTNM classification, however, when re-classified as the Limited and Adequate eLN cohort, significant heterogeneous differences in the 5-year OS were observed between the corresponding substages of the two cohorts, except for stage IB and IIA (Table 3). Also, the 5-year OS of patients from substages IB–IIIB of the Limited eLN cohort was found to approximate those of IIA–IIIC in the Adequate eLN cohort, instead of their corresponding IB–IIIB substages, respectively; which is illustrated by the subsequent overlapping of the survival curves of IB, IIA, IIB, IIIA, IIIB and IIIC of Limited cohort with IIA, IIB, IIIA, IIIB and IIIC of the Adequate cohort respectively (Fig. 3b). Therefore, the nodal subgroups of the AJCC TNM classification were replaced by the N′ nodal subgroups and based on which the TN′M classification was formulated. This reclassification approach showed that the TN′M classification could accommodate a more diversified survival range with superior survival stratification (HR: TN′M, 2.845–34.795 vs. 8th AJCC pTNM, 2.353–20.845). Further, as shown from Fig. 3c, the marked underestimation in the OS of patients from IB to IIIB using the 8th AJCC TNM classification was improved when using the TN′M classification. Lastly, the 5-year OS prognostic performance analysis showed that the TN′M staging system had superior homogeneity, discriminatory ability, and prognostic ability as compared to the 8th AJCC GC edition (Table 4).

## Discussion

In this study, we have concisely demonstrated the significant heterogeneous correlation in survival existing between the corresponding nodal subgroups of the Limited and Adequate eLN cohort when adhering to the customary 8th AJCC pN and pTNM stratification system, which resulted in substantial prognostic difference between these two cohorts of patients. The proposed N′ classification was able to largely compensates for this significant stage migration/misclassification by providing a simple, alternate and more homogenous stratification approach.

The N′ classification also displayed superior stratification and 5-year OS prognostic reliability compared to the two other most studied nodal classification in gastric oncology. Nevertheless, one important hindrance of the N′ classification was that it demonstrated a lower discriminatory ability (lower value of linear trend χ^2^) as compared to the LNR. We hypothesize that this may have been resulted due to the LNR’s algorithm that groups patients with similar nodal ratio irrespective of their total number of LNs retrieved. For instance, patients with 1/10 and 3/30 eLNs (no. of metastasized LNs/total no. of eLNs) would be grouped within the same nodal subgroup if using the LNR but when in fact they are classified as pN1 and pN2, respectively, using the 8th AJCC N classification. Therefore, when such sub-groups were correlated with OS, they tended to merge instead of differentiating their survival differences, thereby resulting in a better discriminatory ability but at the expense of prognostic accuracy. In addition, the LNR has other inherent drawbacks when compared to the N′ classification. First, it tended to demonstrate significant stage migration resulting in an under-/over-estimation of prognoses, particularly for less advanced cases as illustrated by the juxtaposed survival curves in Fig. 2 (N′0 vs. LNR 1, *P* = 0.017). Second, grouping patients with marked differences in overall number of eLNs together within the same group is an analytical bias as these patients have different survival outcomes, making the LNR clinically less reliable and may be one of the contributing factors for its reluctant global acceptance by professional committees such as the AJCC and NCCN.

The AJCC pN classification allows patients’ grouping uniformity in terms of survival by providing a stable stratification method, unlike the LNR, which is hindered by its unstable varying cut-off values between different populations [24, 25], similar populations at different institutions [26, 27], or even similar institutions but with different sample sizes analyzed at different time periods [28, 29]. Furthermore, the LNR and other similar strategies [30, 31] has cumbersome applicability in the daily busy clinical settings as they demand a high level of complicated calculations and do not comply with the easy-to-remember, straightforward AJCC nodal classification criteria. Moreover, it has been demonstrated in an Italian study by Pedrazzani et al. [32]. that the LNR approach has limited utility for patients who have had few eLNs. In contrast, since the N′ classification uses similar stratification analogy to the AJCC N classification, these hurdles are easily overcome, giving it greater potential to be more widely adopted.

Further, multivariate analyses of the N′ classification found that the number of eLNs was not an independent prognostic factor for survival. Also, we noticed that as the prognostic performance of the nodal classification increases, the *P*-value representing its independent correlation with survival increases as well (N, *P* < 0.001; LNR, *P* = 0.041; N′, *P* = 0.374). We, therefore, hypothesize that as the homogeneity in survival between the Limited and Adequate eLN cohort is increased, this decreases the impact of the eLNs category as an independent factor since more emphasis was placed on subgroups’ classification.

Regarding the stage classification, the data from Table 3 show that using the 8th AJCC classification, the 5-year OS of substages IB–IIIB of the Limited eLN cohort approximated those of IIA–IIIC of the Adequate eLN cohort, respectively, with significant intersection observed between their survival curves (all *P* > 0.05). By implementing the TN′M classification, the heterogeneity between these two cohorts was observed to decrease, resulting in significant improvement in the stratification (wider survival range and HR values) and prognostication of the patients (amelioration in the 5-year OS prognostic estimation and superior AIC values). Prior to nodal subgroups re-classification, although there was no significant difference observed between the corresponding substages IB and IIA of the Limited and Adequate eLN cohorts, however, after re-arrangement using the TN′M classification, improved results were obtained as shown by the substantial increase in *P* values from 0.136 to 0.677 and 0.169 to 0.995, respectively. This, therefore, demonstrates an enhanced prognostic estimation approximating to that of the actuarial 5-year OS of the patients (Table 3). Of note, considering that the pT and pM categories were kept constant in all the analyses and that the LNR performance was inferior compared to the N′ classification, for ease of interpretation and avoiding repetition of data, only the prognostic performance for the TN′M and 8th AJCC pTNM classification were provided for this study.

If the stratifying technique proposed in this study can be widely validated, we expect that the proposed novel, optimized and homogenized classification to significantly impact treatment decisions as not only the survival prognostication would be more accurately determined, but most importantly, this approach does not significantly affect the prognostication of patients with Adequate eLNs. Therefore, to a certain extent, we predicate that the proposed classification could facilitate the enrollment of patients, on a more individualized basis, in clinical trials comprised of both categories of patients and improve their obtained results. As an annotation, based on ethical practices, the results of this study are not to be considered for restricting the extent of LN retrieval but, to be used post-gastrectomy for improving the prognostic estimation of patients with limited eLNs, which were due to unexpected circumstances (patient’s or surgical related factors). Thereby, providing oncologists with an unbiased, easy to use, more standardized, and individualized approach for selecting treatment modalities and follow-up evaluations for this category of patients rather than relying on their personal judgment. Subsequently, patients with limited eLNs, especially those staged as IB when using the proposed classification, would have a lesser risk of being under-treated or under-followed.

The limitations of this study are worth mentioning. Patients with early GC could not be analyzed for the following reasons. First, the number of early GC cases were limited in our database as compared to our advanced cases. Second, in our institution, the surgical treatment for patients with T1 disease varied considerably from endoscopic resection (endoscopic submucosal dissection or endoscopic mucosal resection) [33] to D1 and/or D1+ gastrectomy [34] and many of these patients were not treated in our department. Therefore, early GC cases were omitted in the calculations since they could have biased the findings of this study. Second, because of the retrospective and mono-institutional nature of this study, the findings need validation from large multicentered-cohorts and/or in different population settings before being widely applied.

In conclusion, we have developed an easy-to-adopt, reliable and practical stratification approach which uses similar analogy as the 8th AJCC nodal classification to homogeneously classify cohorts of advanced GC patients comprising of both limited and adequate numbers of pathologically eLNs.

## Data Availability

The key raw data have been deposited into the Research Data Deposit (identifier: RDDA2019001026) (http://www.researchdata.org.cn).

## Abbreviations

GC: gastric cancer
LNs: lymph nodes
eLNs: pathologically examined LNs
NCCN: National Comprehensive Cancer Network
AJCC: American Joint Committee on Cancer
SEER: Surveillance, Epidemiology, and End Results Program
pN: pathological nodal classification
OS: overall survival
N′: modified nodal classification
LNR: lymph-node-ratio
TNM: tumor-node-metastasis
TN′M: tumor-modified node-metastasis
T: depth of tumor invasion
AIC: Akaike information criterion values
HR: hazard ratio
CI: confidence interval
